# Supplementary figures and images for: Harnessing Genetic Diversity in the USDA Pea Germplasm Collection Through Genomic Prediction
Source: Front Genet. 2021 Dec 24;12:707754. doi: 10.3389/fgene.2021.707754 (PMC8740293; doi:10.3389/fgene.2021.707754)

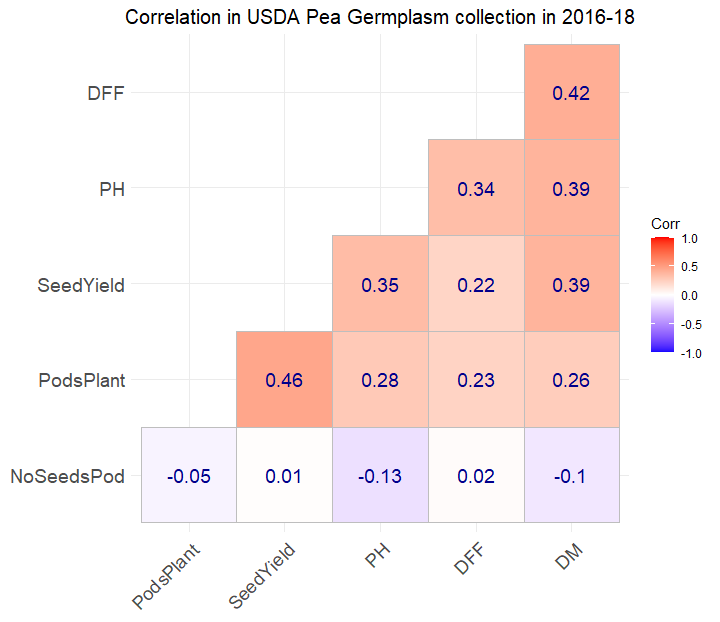

Supplement: Supplementary file 2 [file Image1.tiff]

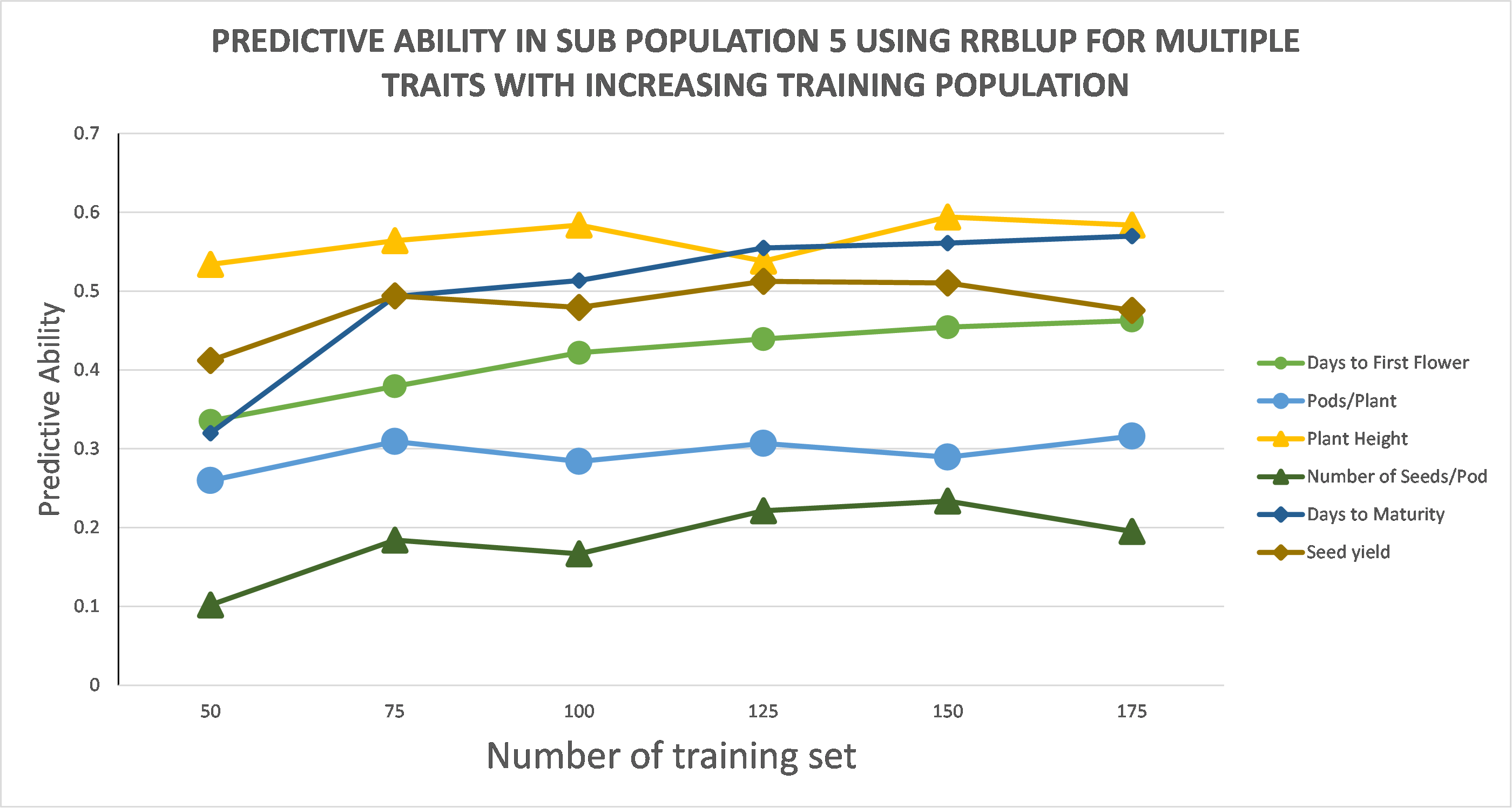

Supplement: Supplementary file 5 [file Image3.tif]

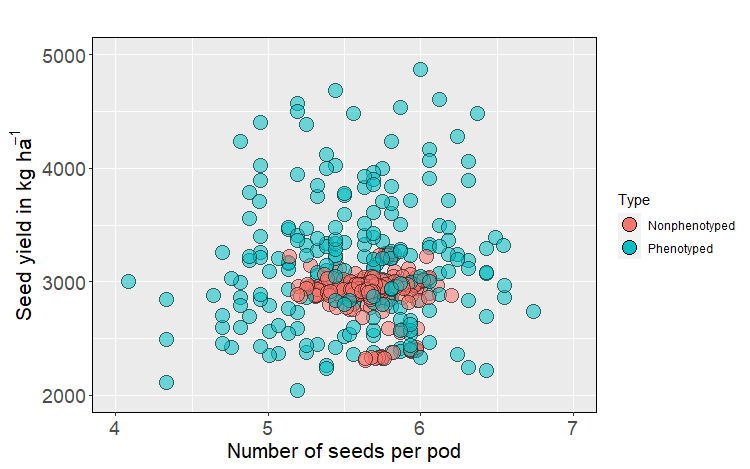

Supplement: Supplementary file 6 [file Image5.tiff]

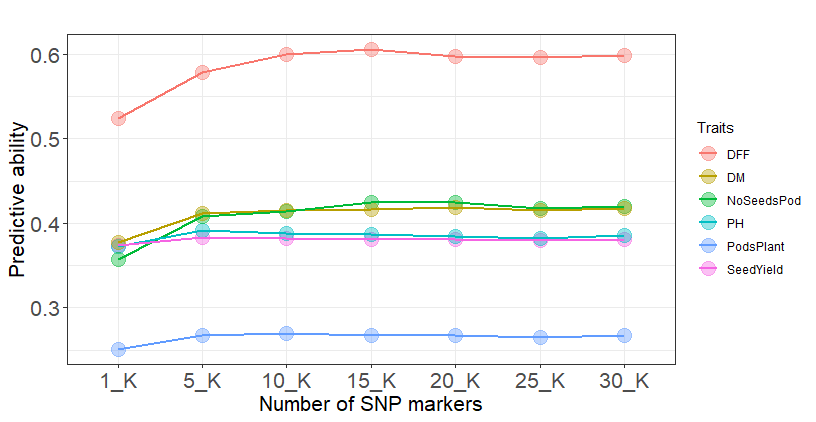

Supplement: Supplementary file 11 [file Image2.tiff]

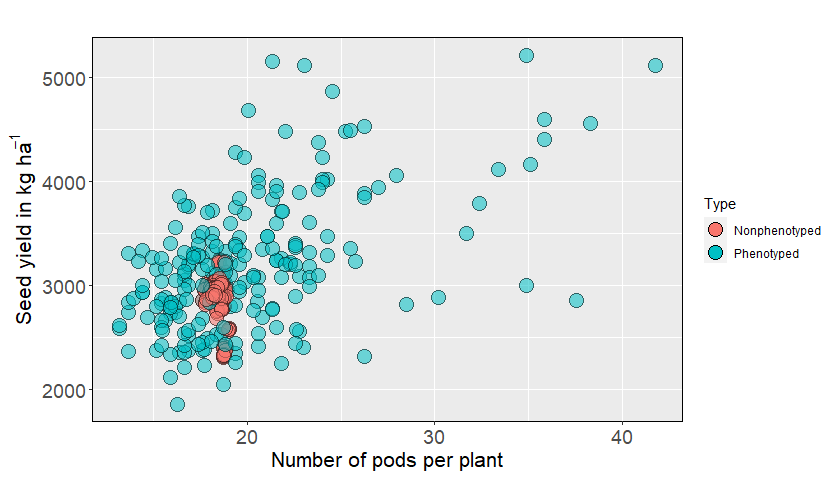

Supplement: Supplementary file 12 [file Image4.tiff]
